# Efficacy and safety of mycophenolate mofetil in the treatment of moderate to severe Graves’ orbitopathy: a meta-analysis

**DOI:** 10.1080/21655979.2022.2101191

**Published:** 2022-08-12

**Authors:** Wenwen Feng, Yifang Hu, Chengzhou Zhang, He Shi, Peng Zhang, Yanli Yang, Shiying Chen, Weijiang Cui, Dai Cui

**Affiliations:** a Department of Endocrinology, the First Affiliated Hospital of Nanjing Medical University, Nanjing, China; b Department of Geriatric Endocrinology, the First Affiliated Hospital of Nanjing Medical University, Nanjing, China; c Department of Internal Medicine, Maternal and Child Health Hospital of Yancheng, Yancheng, China; d Department of Nuclear Medicine, the First Affiliated Hospital of Nanjing Medical University, Nanjing, China; e Department of Ophthalmology, Ili & Jiangsu Joint Institute of Health, the Friendship Hospital of Ili Kazakh Autonomous Prefecture, Ili, China

**Keywords:** Mycophenolate mofetil, Graves’ orbitopathy, meta-analysis, treatment

## Abstract

The role of mycophenolate mofetil (MMF) in the treatment of Graves’ orbitopathy (GO) has attracted much attention. This study is to evaluate the benefit and safety of MMF in moderate-to-severe GO. A meta-analysis of clinical control trials comparing MMF (with or without glucocorticoid (GC)) for the treatment of GO with GC was conducted. We searched the databases, including PubMed, EMBASE, the Cochrane Library, Web of Science, Wanfang, and China National Knowledge Infrastructure (CNKI), for articles published up to 15 June 2022. The primary outcome is referred to the improvement in overall response, and secondary outcomes included the change in clinical activity score (CAS) and adverse events (AEs). Of the 289 articles initially searched, 6 studies were finally eligible for inclusion. The results showed that MMF (with or without GC) was superior to GC in the treatment of GO (OR 3.34, 95% CI 2.17–5.14; *p < *0.00001). Subgroup analyses also showed that MMF monotherapy was more effective than GC (OR 4.46, 95% CI 2.52–7.87; *p < *0.00001). Compared to methylprednisolone (MP) monotherapy, a combination of MP and MMF was more effective. CAS decreased even more significantly (WMD 0.29, 95% CI 0.10–0.48; *p* = 0.002) and fewer AEs occurred (OR 0.2, 95% CI 0.06–0.72; *p = *0.01) in patients receiving MMF. The pooled data suggested that MMF treatment in GO might be promising. Compared with GC therapy, MMF is safer and more effective. However, more large-sample and high-quality studies targeting MMF use in GO patients and long-term surveillance of prognosis are urgently needed.

## Graphical Abstract

**Figure:**
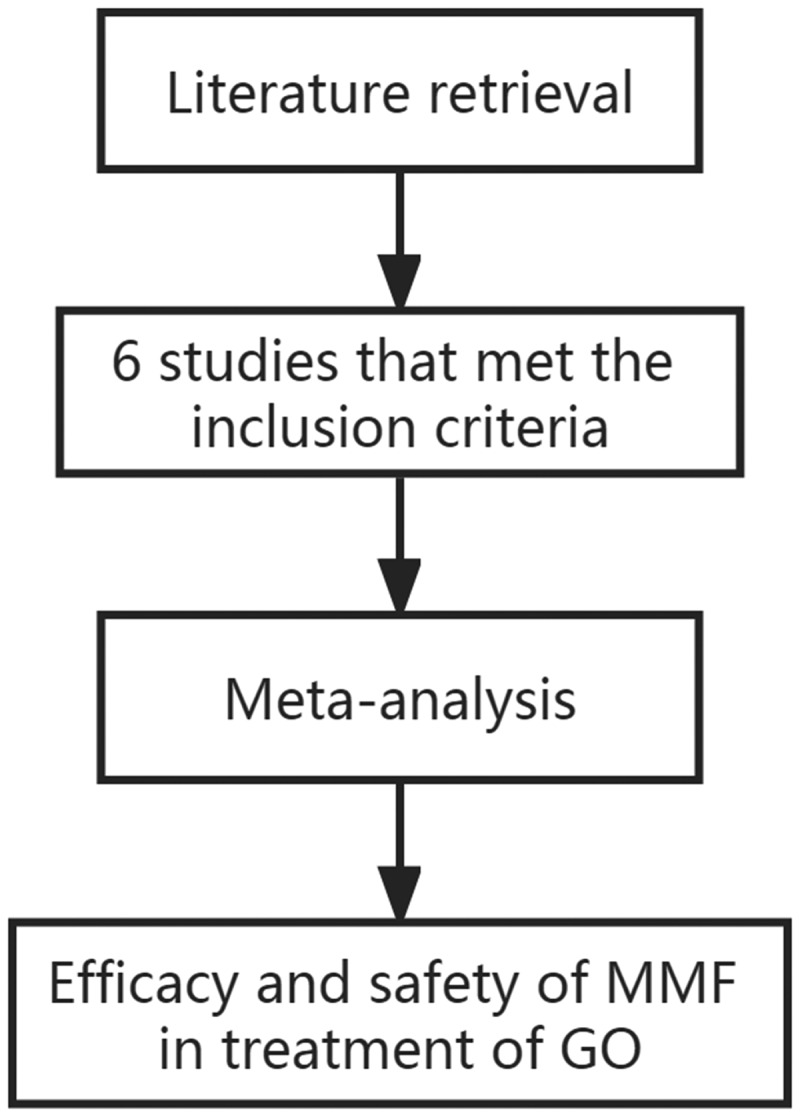

## Highlights

- MMF is more effective in the treatment of GO than glucocorticoid therapy.
- Compared with glucocorticoid treatment, the decrease in CAS score was more significant in MMF treatment for GO.
- MMF treatment for GO has fewer adverse reactions compared with glucocorticoid therapy.

## Introduction

Graves’ orbitopathy (GO) is the main extrathyroidal manifestation of autoimmune thyroid disease. It occurs in 30–50% of Graves’ disease (GD) patients. About 5% of patients are moderate-to-severe cases, which can lead to vision loss and even blindness, resulting in long-term disability [1]. The pathogenesis of GO has not been fully clarified yet. Currently, the T cell-mediated immunity is considered to be the main course of GO onset [2,3]. The treatment of GO is still a challenge for some patients. Currently, glucocorticoids (GCs) are the mainstay of treatment. Weekly intravenous methylprednisolone (IVMP) injection for 12 weeks is the most widely used first-line treatment for active moderate-to-severe GO, i.e. 0.5 g per week for the first 6 weeks and 0.25 g per week for the next 6 weeks [4]. However, recurrence often occurs once GCs are withdrawn [5]. Besides, the use of GC may result in a greater frequency of adverse events (AEs), such as imbalance of glucose metabolism, electrolyte disorder, liver damage, and cardiovascular or cerebrovascular events. In recent years, researchers have proven that many immunosuppressants such as mycophenolate and azathioprine as well as biological agents such as teprotumumab and tocilizumab are effective for GO [6].

Mycophenolate mofetil (MMF), as an immunosuppressant, has been used for organ transplantation and several autoimmune diseases [7–9]. MMF is a prodrug of mycophenolic acid (MPA), which depletes guanosine triphosphate and suppresses the *de novo* synthesis of purines, thus inhibiting proliferation and inducing apoptosis of activated T lymphocytes [10]. European Group on Graves’ Orbitopathy (EUGOGO) recommended that IVMP combined with MMF could be used as a first-line treatment for moderate-to-severe and active GO based on long-term efficacy, safety, cost, availability, and patient selection [11].

This meta-analysis aims to systematically summarize the efficacy and safety of MMF in the treatment of moderate-to-severe GO. Compared with classical GC therapy, the superiority of MMF in the treatment of GO was analyzed, so as to provide more treatment options for GO in clinic.

## Methods

This meta-analysis was conducted and reported according to the Preferred Reporting Items for Systematic Reviews and Meta-Analyses (PRISMA) statement [12].

### Search Strategy

Through the databases, including PubMed, EMBASE, the Cochrane Library, Web of Science, China National Knowledge Infrastructure (CNKI), and Wan Fang, a systematic search for all published literature that evaluated the efficacy and safety of MMF in the treatment of GO was conducted. The last search was performed on 15 June 2022. The following search terms were used: ‘Graves’ Ophthalmopathy,’ ‘Graves’ orbitopathy,’ ‘thyroid-associated ophthalmopathy,’ ‘thyroid eye disease,’ ‘Mycophenolate Mofetil,’ ‘Mycophenolate,’ and ‘MMF.’ Besides, we identified other studies by searching the reference sections of the relevant articles and collected the studies that matched the inclusion criteria. The three authors searched all literature independently. All existing contradictions were resolved through discussion.

### Outcome measures

The primary outcome is referred to the improvement in overall response. ‘Response’ can be defined as soft tissue changes, pain, proptosis, eye movement function, eye muscle involvement, and vision. The secondary efficacy outcome included the change in clinical activity score (CAS) [4,13]. Adverse events (AEs) were also assessed, including gastrointestinal events, liver function damage, menstrual disorder, Cushingoid symptoms, infection, hypertension, and hyperglycemia.

### Selection criteria

Selected studies met the following criteria: (1) Study type: a prospective or retrospective cohort study in English or Chinese language. (2) Population: GO patients who have not been treated with GCs or immunosuppressive therapy, meeting CAS ≥3 or NO SPECS ≥2. (3) Intervention: Oral MMF, with or without the combined therapy, versus GC therapy. (4) Outcome variables: one or more of the outcome variables be covered, including the response, the change in CAS, and the occurrence of AEs.

All the excluded studies met the following criteria: (1) case reports, reviews, letters, animal studies, or no insufficient data; (2) without control group; and (3) full text unavailable.

### Data extraction and quality assessment

Data extraction was performed according to the defining rules from each study [10,14–18], independently by three reviewers. The contents of collected data included in this meta-analysis are listed as follows: (1)Method: the randomization method, control group, double or single blindness, the number of the lost follow-up. (2) Participants: the inclusion and exclusion criteria, sample size, patient sex, age, disease course, activity, and severity of GO. (3) Interventions: route, dose, co-interventions, comparison interventions, and duration of interventions. (4) Outcomes: efficacy outcomes (overall response and the change in CAS) and adverse events. (5) Notes: General information on the literature, such as article source, title, authors, and published year. According to the Cochrane Collaboration’s tool [19], the quality of the studies was assessed. The Cochrane Collaboration’s tool describes seven parameters, including random sequence generation, allocation concealment, blinding of participants and personnel (performance bias), blinding of outcome assessment (detection bias), incomplete outcome data (attrition bias), selective reporting (reporting bias), and other biases. Among them, random sequence generation and allocation concealment are used to assess selection bias. Through the evaluation, each parameter was graded as low risk, unknown risk, and high risk.

### Statistical analysis

The statistical analysis was performed using the Review Manager software version 5.4 from the Cochrane Collaboration. For efficacy outcomes, odds ratios (ORs) with 95% confidence intervals (CIs) for dichotomous outcomes and weighted mean difference (WMD) with 95% CIs for continuous outcomes were calculated.

The statistical heterogeneity was assessed by Cochran’s Q statistic and *I*^2^ metrics [20]. When the *p*-value >0.05 and *I*^2^ value <50%, the analysis was considered non-heterogeneous, and the fixed-effects model was used for meta-analyses. Otherwise, it was considered to be heterogeneous, and the random-effects model was used. The heterogeneity was also explored by conducting subgroup analyses.

Subgroup analyses were conducted based on a single drug or combination therapy. Standard funnel plots were also constructed to evaluate the potential publication bias, by examining visually the asymmetry. The sensitivity analyses were conducted through the Stata software version 16.

## Results

### Brief introduction

MMF treatment for moderate-to-severe GO has attracted more and more attention. We searched PubMed, EMBASE, the Cochrane Library, Web of Science, CNKI, and Wan Fang databases for all published literatures on the efficacy and safety of MMF for GO and conducted meta-analyses. The results showed that compared with GC monotherapy, MMF had a higher response rate, better efficacy, and fewer AEs in moderate-to-severe GO, either monotherapy or in combination with GC.

### Study characteristics

**Figure 1.**
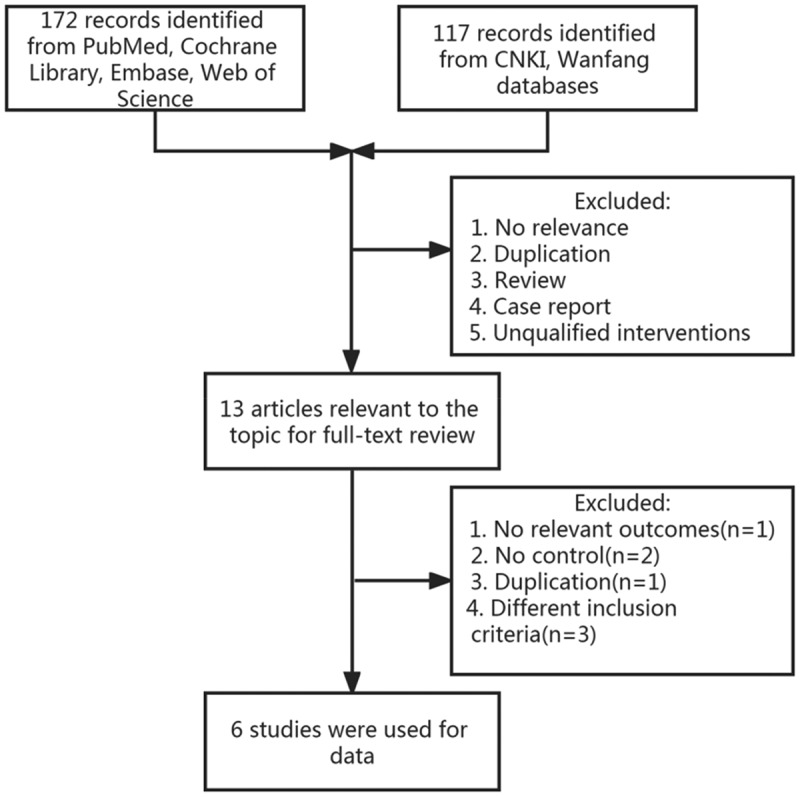
Flow diagram of study selection.

A total of 289 articles were obtained from six databases. Thirteen studies were recruited for further full-text reading after reading the title and abstract, of which 6 articles were finally available for inclusion. The selection flow is shown in Figure 1. The basic characteristics of the included studies are presented in Table 1. It can be found that the participants in this study were from 12 to 75 years old. The population consisted of 219 (36.6%) men and 379 (63.4%) women and 2.6% were from China.

**Table 1.** Baseline characteristics of eligible clinical trials.

Study	Location	Patients (*n*)	Age (years)	Sex (M/F)	Disease severity	Course of the disease	Intervention	Control
Wang 2003	China	51	21–54	24/27	NOSPECS ≥2 levels	1–12 months	MMF/PO, 1 g/day for 12 weeks (12)	Prednisone/PO, 40 mg/day for 4 weeks 20 mg/day for 4 weeks; 10 mg/day for 4 weeks (13)
Zhang 2011	China	70	21–63	48/22	NOSPECS ≥2 levels	MMF: 6–32 months; MP: 1–15 months	MMF/PO, 1.5 g/day for 8 weeks; 0.5–0.75 g/day for 3–6 months (meanwhile, prednisone/PO, 1 mg/kg/day, reduce to a maintenance level, last for 3–6 months (35)	MP/IVD, 0.5 g/day * 3 days/week for 4 weeks Prednisone/PO, 1 mg/kg/day, reduce to a maintenance level, last for 3–6 months (35)
Cui 2013	China	115	Dec-70	38/77	NOSPECS ≥2 levels	1 week–20 years	MMF/PO, 1 g/day for 12 weeks (40)	Prednisone/PO, 40 mg/day for 4 weeks 20 mg/day for 4 weeks; 10 mg/day for 4 weeks (30)
Hu 2015	China	40	25–45	18/22	NOSPECS ≥2 levels; CAS ≥3 scores (3–7)	No	MMF/PO, 1 g/day for 12 weeks (22)	Prednisone/PO, 40 mg/day for 4 weeks 20 mg/day for 4 weeks; 10 mg/day for 4 weeks(18)
Ye 2017	China	158	16–70	52/106	Moderate-to-severe; CAS ≥4 scores (4–10)	MMF: 5.84 ± 3.71 months GC: 6.37 ± 5.89 months	MMF/PO, 1 g/day for 24 weeks (80)	MP/IVD, 0.5 g/day * 3 day/week for 2 weeks; Prednisone/PO, 60 mg/day * 8 weeks a reduction of 5 mg per week for 14 weeks (78)
Kahaly 2018	Germany, Italy	164	18–75	39/125	Moderate-to-severe; CAS ≥3 scores (3–7)	MP + MMF: 5–19.5 months MP: 4–18 months	MP/IVD, 0.5 g/week for 6 weeks; 0.25 g/week for 6 weeks. MMF/PO, 0.72 g/day for 24 weeks(75)	MP/IVD, 0.5 g/week for 6 weeks 0.25 g/week for 6 weeks (72)

Abbreviations: MMF = mycophenolate mofetil; PO = per os; MP = methylprednisolone; IVD = intravenous drip; GC = glucocorticoid; CAS = clinical activity score; wks = weeks

### Risk of bias and publication bias

**Figure 2.**
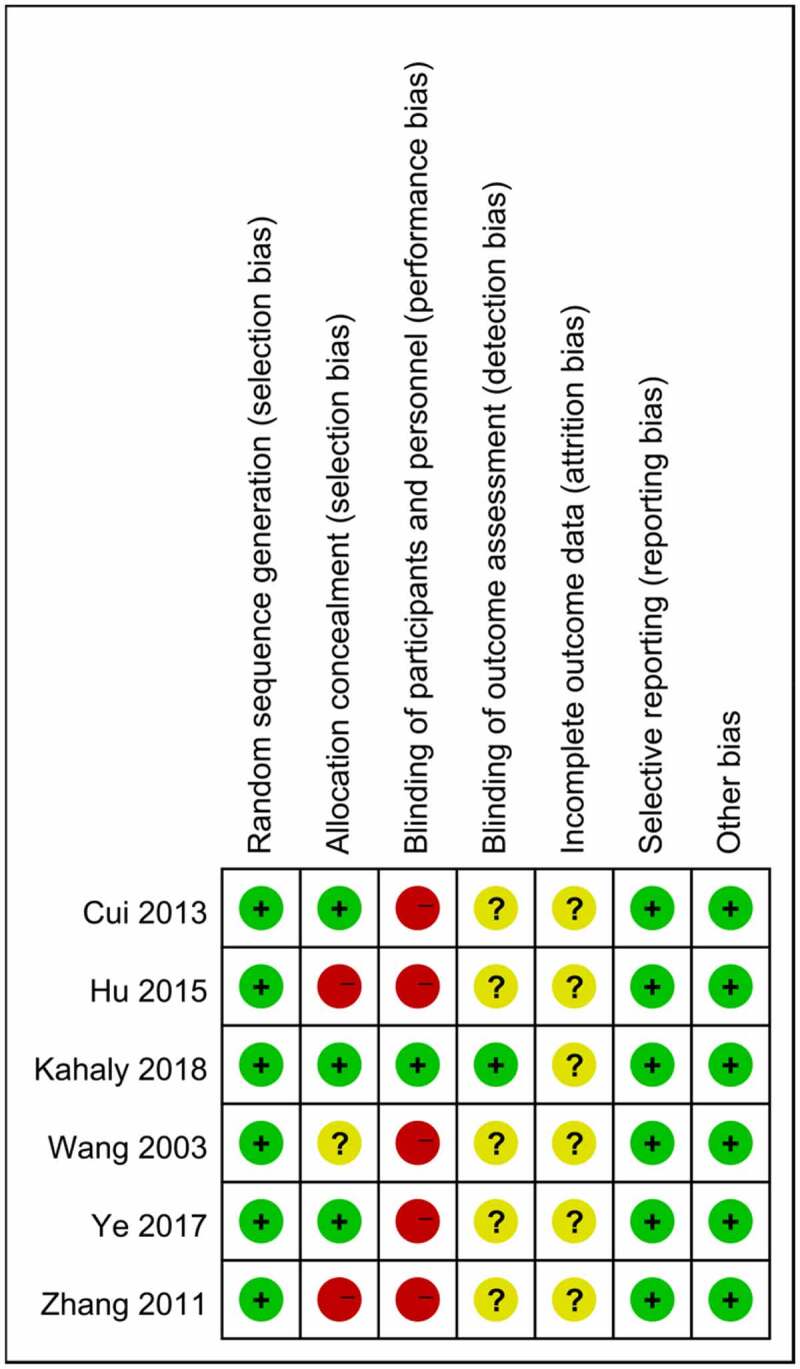
Risk of bias of included studies.

**Figure 3.**
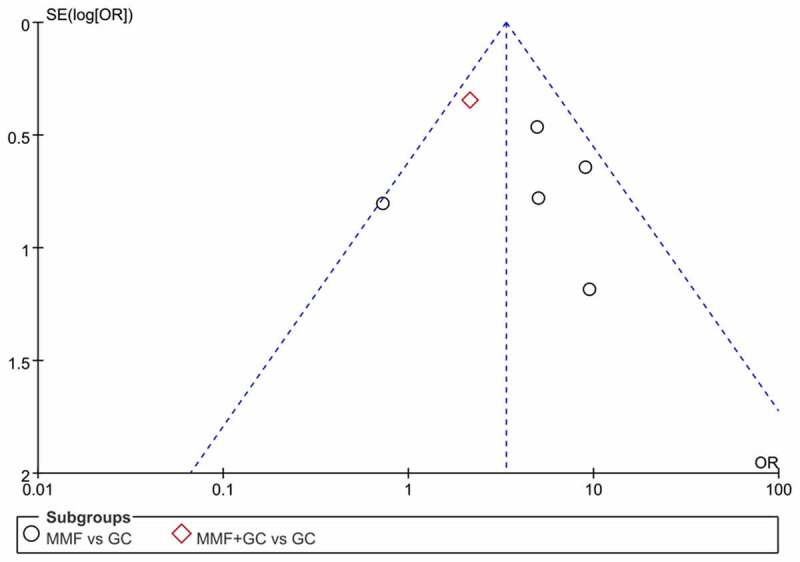
Funnel plot for the response of MMF (with or without GC) versus GC. MMF: mycophenolate mofetil; GC: glucocorticoid.

Through the Cochrane Collaboration tool, two trials had a high risk of bias in two domains of selection bias and performance bias, and three trials had a high risk of bias in one domain of performance bias. One trial was found with a low risk of bias (Figure 2). Funnel plot for the response of MMF (with or without GC) versus GC is qualitatively symmetrical, indicating a low probability of publication bias (Figure 3).

### Sensitivity analyses

**Figure 4.**
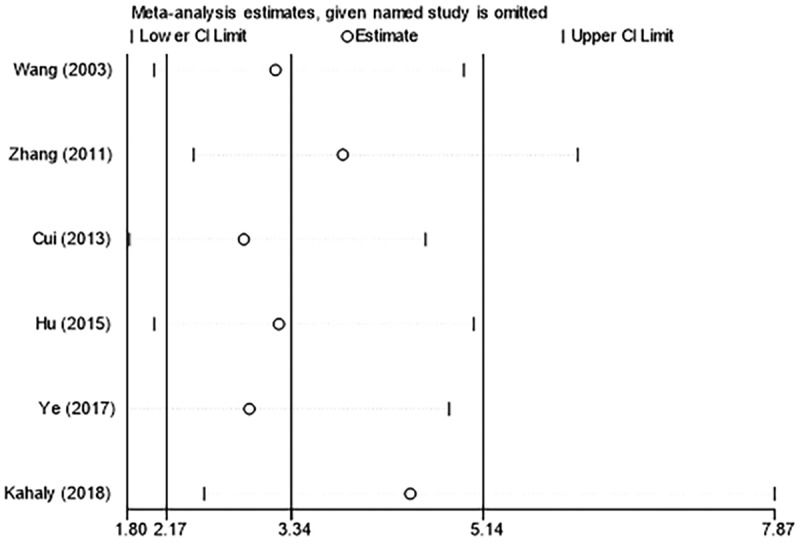
Sensitivity analysis for the response of MMF (with or without GC) versus GC. MMF: mycophenolate mofetil; GC: glucocorticoid.

Sensitivity analyses were carried out for the six included studies by eliminating the literature one by one. The results showed that the combined OR had high stability, which also indicated that the data had high consistency and reliability (Figure 4).

### Data analysis

**Figure 5.**
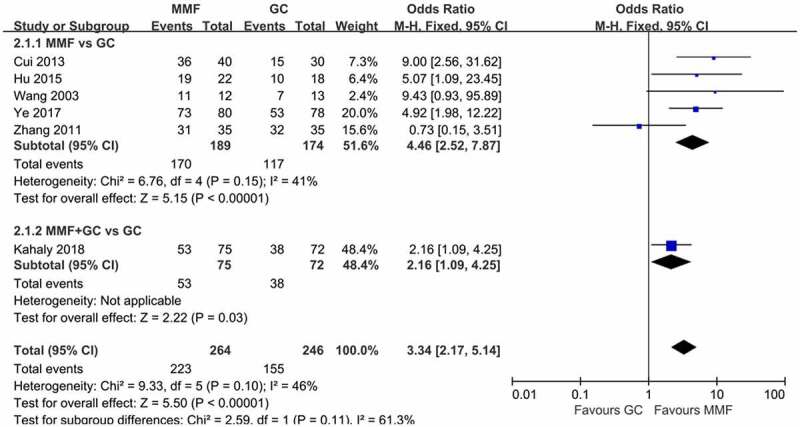
Forest plot for the response of MMF (with or without GC) versus GC. MMF: mycophenolate mofetil; GC: glucocorticoid.

The forest plot of response comparing MMF with GC is shown in Figure 5. A total of six articles were included in this meta-analysis, five of which compared the overall efficacy of MMF and GC alone in the treatment of GO, and one compared the efficacy of MMF combined with GC and GC alone in the treatment of GO. The results of the six literature were combined, and the heterogeneity results suggested that there was no heterogeneity among the studies (*I*^2^ = 46%, *p > *0.05), so the fixed-effects model was used for the combined analysis. The results showed that MMF (with or without GC) was superior to GC in the treatment of GO (OR 3.34, 95% CI 2.17–5.14; *p < *0.00001). Subgroup analysis showed that MMF monotherapy was superior to GC (OR 4.46, 95% CI 2.52–7.87; *p < *0.00001).

**Figure 6.**
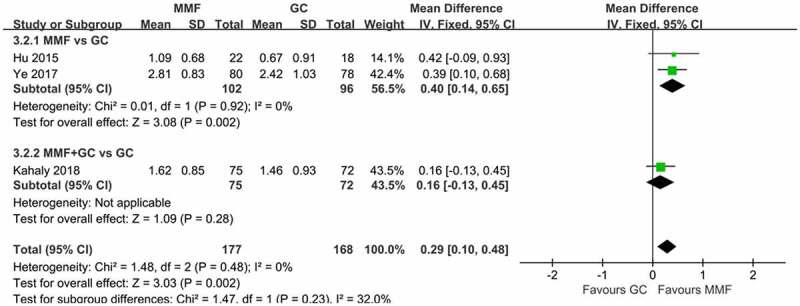
Forest plot for CAS change of MMF (with or without GC) versus GC. CAS: clinical activity score; MMF: mycophenolate mofetil; GC: glucocorticoid.

The severity of eye disease was evaluated by NO SPECS grading standards set by the American Thyroid Association [21] and CAS. Three articles described the CAS score of patients at 12 weeks of drug therapy. The reduction of CAS before and after treatment were analyzed (Figure 6). The heterogeneity results indicated that there was no heterogeneity among the studies (*I*^2^ = 0%, *p* = 0.48), so the fixed-effects model was used for the combined analysis. The results showed that MMF (with or without GC) significantly reduced CAS scores in GO patients compared with GC treatment (WMD 0.29, 95% CI 0.10–0.48; *p* = 0.002).

**Figure 7.**
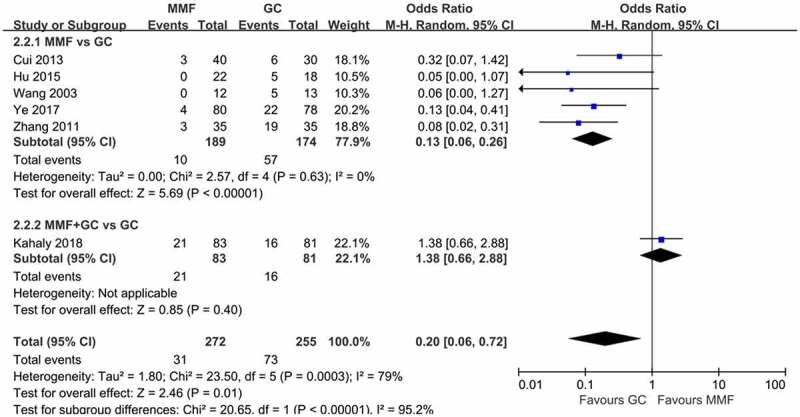
Forest plot for the incidence of AE of MMF (with or without GC) versus GC. AE: adverse event; MMF: mycophenolate mofetil; GC: glucocorticoid.

The incidence of AEs was used as a safety indicator in this study. The six included articles reported AEs in GO treatment with MMF and GC. MMF group had significantly fewer AEs. In two of the six studies, there were no AEs in GO patients treated with MMF. In Zhang’s study [15], three cases of gastrointestinal symptoms occurred in the MMF group. Three cases of elevated transaminases were reported in Cui’s article [16], and four cases of AEs were reported in Ye’s article [10], including one case of hypokalemia and three cases of mild liver dysfunction. MMF combined with GC produced more AEs than did GC monotherapy in Kahaly’ study [18]. The incidence of AEs in all studies was meta-analyzed (Figure 7). Heterogeneity test results indicated that there was heterogeneity among studies (*I*^2^ = 79%, *p* = 0.003), so the random-effects model was used for the combined analysis. The results showed that fewer AEs occurred with MMF compared with GC for GO (OR 0.2, 95% CI 0.06–0.72; *p* = 0.01).

## Discussion

GO is an organ-specific autoimmune disease associated with Graves’ disease (GD), which potentially causes vision loss, disfigurement, and increased pain and severely affects the quality of life of patients [22]. The pathogenesis of GO is still unclear, and it is currently believed that the T cell-mediated immunity has an important role in orbital inflammation in GO. A large number of T cells, mainly CD4+ T cells, were infiltrated in orbital tissues of GO patients. T cells are activated by autoantigens and interact with orbital fibroblasts through specific receptor–ligand bridges and transduction of a series of intracellular cascade signals, promoting fibroblast proliferation, hyaluronan secretion, adipogenesis, and inflammatory factor production. Orbital fibroblast surface receptors, including thyrotropin receptor and insulin-like growth factor-1 receptor, are closely related to these processes [23]. This results in periorbital inflammation edema, protrusion, and other ocular symptoms. Smoking, gender, thyroid dysfunction, and high serum thyrotropin receptor antibody level also have varying degrees of influence on the incidence of GO.

Some treatments are common for patients with active mild GO, such as risk factor control, topical treatment, and selenium supplementation. For patients with active moderate-to-severe and sight-threatening GO, intravenous GC injection is the most common in addition to antithyroid drugs to control hyperthyroidism. However, after intravenous injection of large doses of methylprednisolone (MP), some adverse events occur frequently, such as hypertension, abnormal glucose metabolism, stress ulcer, and electrolyte disorder. In severe cases, acute heart failure, acute myocardial infarction, autoimmune hepatitis, and fatal liver failure can occur [24]. In addition, the overall response rate of GC therapy in GO patients is only 50–60%. Therefore, it is necessary to explore other potential treatments to improve the clinical symptoms and signs of GO patients and improve patients’ quality of life. As a new immunosuppressant, MMF has attracted a lot of attention for its special effect on lymphocytes and its safety on the heart, liver, and kidney.

MMF is the precursor of MPA, which is widely used in organ transplantation [25] and autoimmune disease [26,27]. MPA is an inhibitor of inosine monophosphate dehydrogenase. This is the rate-limiting enzyme in *de novo* synthesis of guanosine nucleotides. MPA causes depletion of guanosine-triphosphate and ultimately apoptosis of activated T-lymphocytes because lymphocytes produce guanosine nucleotides mainly through the *de novo* synthesis pathway rather than the salvage pathway. MMF can be hydrolyzed by esterases to form the active metabolite MPA. This is the principal mechanism for MMF exerting an immunosuppressive effect [28]. Compared with other cell types, it has a stronger inhibitory effect on T lymphocytes and seldom damages non-lymphocyte organs. In addition, it has no significant reproductive toxicity and hepatorenal toxicity, which is its advantage. However, it also has some adverse events, such as gastrointestinal reaction, infection, and electrolyte disturbance [29].

This meta-analysis evaluated the efficacy and safety of MMF in the treatment of GO compared with GC monotherapy. The results showed that MMF had a higher response rate in active moderate-to-severe GO, either monotherapy or in combination with intravenous GC. Subgroup analysis suggested that MMF monotherapy was superior to GC monotherapy, and MMF combined with GC therapy was also superior to GC monotherapy. In addition, our study found that MMF reduced CAS more significantly in GO patients. It has been reported that MMF combined with low-dose prednisolone therapy could be introduced as a new optimal administration for GO due to its advantages in chronic complications such as proptosis and diplopia [30]. Finally, our results also showed that fewer AEs occurred with MMF compared to GC therapy. Riedl et al. analyzed the AEs of moderate-dose MMF in GO patients, and the results showed that MMF was well tolerated [31]. Currently, MMF is mostly used to treat patients with active moderate-to-severe GO who are resistant to GC, and some studies indicate that MMF is safe and effective as a second-line immunosuppressant [32,33]. The 2021 EUGOGO recommended intravenous MP in combination with MMF as a first-line treatment, taking all factors into account. However, Kahaly et al. reported that compared with intravenous MP alone, drug combination has a higher incidence of AEs [18]. Therefore, MMF monotherapy for GO needs more clinical trials because of its good efficacy and safety.

We conducted a meta-analysis of the latest research on MMF in the treatment of GO to help guide clinical practice. However, this study also has some limitations. First, the groups were not completely randomized. Some groups were divided according to the patients’ wishes and lacked objectivity. Second, baseline clinical data were inconsistent, such as the course of the disease. Third, the classification of GC in the control group was inconsistent, and the use method was inconsistent. Some control groups took prednisone orally and others received intravenous MP. Lastly, the research sample is too small. We need more multi-centralized, randomized, controlled, and double-blind trials to confirm our results.

## Conclusions

This study found that compared with GC monotherapy, MMF had a higher response rate, better efficacy, and fewer AEs in active GO, either monotherapy or in combination with GC. In the future, more multi-centralized, randomized, controlled, and double-blind trials are needed to confirm our results.

## Data Availability

All data are provided in this article, and data processing methods can be obtained from corresponding author upon reasonable request.
